# Supplementary figures and images for: Endoglin and Other Angiogenesis Markers in Recurrent Varicose Veins
Source: J Pers Med. 2022 Mar 25;12(4):528. doi: 10.3390/jpm12040528 (PMC9025299; doi:10.3390/jpm12040528)

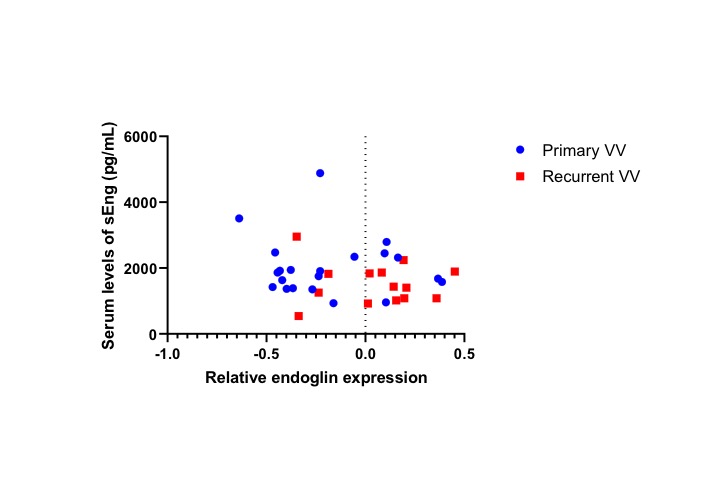

Supplement: Supplementary file 1 [file jpm-12-00528-s001.zip › jpm-1600095-Supplementary Figure S1.jpeg]
